# Exploring the RNA Editing Events and Their Potential Regulatory Roles in Tea Plant (*Camellia sinensis* L.)

**DOI:** 10.3390/ijms232113640

**Published:** 2022-11-07

**Authors:** Mengyuan Zhang, Zhuo Li, Zijian Wang, Yao Xiao, Lu Bao, Min Wang, Chuanjing An, Yuefang Gao

**Affiliations:** 1 College of Horticulture, Northwest A&F University, Yangling, Xianyang 712100, China; 2 College of Language and Culture, Northwest A&F University, Yangling, Xianyang 712100, China; 3 College of Food Science and Engineering, Northwest A&F University, Yangling, Xianyang 712100, China; 4 State Key Laboratory of Natural and Biomimetic Drugs, Department of Chemical Biology, School of Pharmaceutical Sciences, Peking University, Beijing 100191, China

**Keywords:** RNA editing, pentatricopeptide repeat (PPR), albino, etiolation, tea plant

## Abstract

RNA editing is a post-transcriptional modification process that alters the RNA sequence relative to the genomic blueprint. In plant organelles (namely, mitochondria and chloroplasts), the most common type is C-to-U, and the absence of C-to-U RNA editing results in abnormal plant development, such as etiolation and albino leaves, aborted embryonic development and retarded seedling growth. Here, through PREP, RES-Scanner, PCR and RT-PCR analyses, 38 and 139 RNA editing sites were identified from the chloroplast and mitochondrial genomes of *Camellia sinensis*, respectively. Analysis of the base preference around the RNA editing sites showed that in the −1 position of the edited C had more frequent occurrences of T whereas rare occurrences of G. Three conserved motifs were identified at 25 bases upstream of the RNA editing site. Structural analyses indicated that the RNA secondary structure of 32 genes, protein secondary structure of 37 genes and the three-dimensional structure of 5 proteins were altered due to RNA editing. The editing level analysis of *matK* and *ndhD* in six tea cultivars indicated that *matK-701* might be involved in the color change of tea leaves. Furthermore, 218 PLS-CsPPR proteins were predicted to interact with the identified RNA editing sites. In conclusion, this study provides comprehensive insight into RNA editing events, which will facilitate further study of the RNA editing phenomenon of the tea plant.

## 1. Introduction

RNA editing is a post-transcriptional process that modifies the nucleotide sequence of RNA [1]. During editing events, information from genomic DNA (gDNA) to the RNA and protein is concomitantly trimmed [2]. RNA editing events can be divided into nucleotide conversion and nucleotide deletion/insertion. Nucleotide conversion has three main forms [2]: cytosine (C) to uracil (U), uracil (U) to cytosine (C) and adenosine (A) to inosine (I). In plants, C-to-U conversion is the most prevalent form [3], while U to C transitions have only been reported in a few species, such as ferns, lycophytes and hornworts [4]. A to I editing commonly occurs in introns, as well as in the 5′ and 3′ untranslated regions (UTRs) of RNA sequences in a few mammals [2,5]. Nucleotide deletion/insertion mainly occurs at U and guanosine (G) [2,6].

RNA editing of C-to-U was first reported in the mitochondria of flowering plants [7,8,9], followed by in plant chloroplasts [10]. C-to-U conversion seems to occur only in these two energy-producing organelles [11]. In addition to the coding sequence (CDS) of messenger RNA (mRNA), C-to-U editing events occur in ribosomal RNA (rRNA) and transfer RNA (tRNA), as well as in the intron sequences and 5′ and 3′ UTRs of mRNAs [2]. Most C-to-U editing sites are located at the first or second position of a codon and can alter the codons by introducing a new initiation (ACG to AUG) or stop codon (CGA to UGA, CAA to UAA), which might result in amino acid substitution or extension/shortening of the open reading frame [11]. The absence of C-to-U RNA editing can cause abnormal plant development, reflected in etiolation [12], albino [13] or yellow leaves [14], aborted embryonic development [15] and retarded seedling growth [16]. Benefitting from the next generation sequencing technology, genome-scale analysis of C-to-U editing events was extensively studied in various plants [17], including *Arabidopsis thaliana* [18], *Triticum monococcum* [19], *Zea mays* [20] and *Salvia miltiorrhiza* [21]. However, the relationship between RNA editing events and the observed phenotypes in mutants remains unelucidated.

RNA editing of C-to-U is a deamination reaction [22]. A number of RNA editing factors were found to be involved in this process [3], including PLS-type pentatricopeptide repeat (PPR) family proteins [23], multiple organellar RNA editing factor (MORF, also named RNA editing factor interacting protein (RIP)) family proteins [24,25], organelle RNA recognition motif-containing (ORRM) family proteins [26], protoporphyrinogen IX oxidase 1 (PPO1) [27], Chlororespiratory reduction 4 (CRR4) [28] and organelle zinc finger 1 (OZ1) [29]. Numerous reports demonstrated that PLS-PPR proteins are involved in the C-to-U deamination process in chloroplasts and mitochondria [3,23]. PLS-PPR proteins also play crucial roles in chlorophyll accumulation [30] and the development of chloroplasts [30] and mitochondria [31]. Comprehensive analysis of RNA editing factor mutants will contribute to our understanding of the phenotypes observed in these mutants.

In China, many albino and etiolation tea cultivars, such as ‘Baiye 1’, ‘Huangjinya’, ‘Huabai 1’ and ’Baijiguan’ [32,33,34], attract more attention due to their umami taste, lower astringency and higher economic and ornamental value [35]. Numerous studies reported that loss of the RNA editing function could lead to changes in plant leaf color [12,30]. In our previous study, in which the PPR family proteins were systematically analyzed, 295 PLS-CsPPR subfamily members were found in the tea plant [36]. However, there are few reports on the regulatory role of RNA editing in tea plants, and it is unclear whether PLS-CsPPR proteins are involved in the RNA editing process. In this report, RNA editing sites in the chloroplast and mitochondrial genomes of *C. sinensis* were predicted and partially validated. The base preference and potential motifs of sequences surrounding the editing sites were investigated. RNA and the protein secondary structure, protein transmembrane structure domain and protein three-dimensional (3-D) structure were predicted from the sequences of RNA or protein before and after RNA editing. The editing level of *matK* and *ndhD* in six tea cultivars were analyzed. In addition, the interaction between PLS-CsPPR proteins and RNA editing sites was analyzed. Our results are helpful to further study of the RNA editing in the tea plant, as well as provide a theoretical and experimental basis for the breeding of albino and etiolation tea varieties.

## 2. Results

### 2.1. Identification of RNA Editing Sites in the Chloroplast and Mitochondrial Genomes of Tea Plants

**Figure 1:**
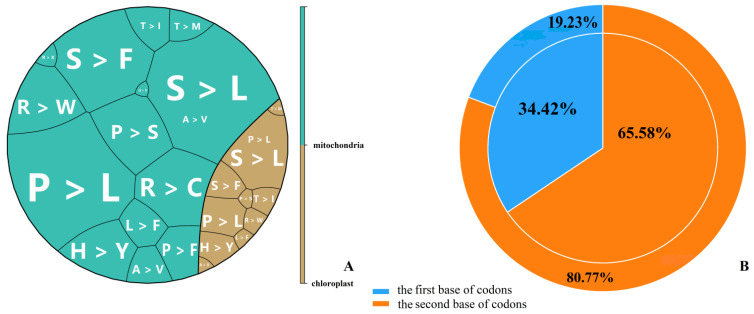
RNA editing sites predicted by PREP suite in tea chloroplast and mitochondria. (**A**) Amino acid residue substitutions resulting from RNA editing; the letters are the abbreviations for amino acid residues, and the block size represents the number of RNA editing sites. (**B**) Position of RNA editing sites in codons. The outer pie charts represent chloroplasts, and the inner pie charts represent mitochondria.

**Figure 2:**
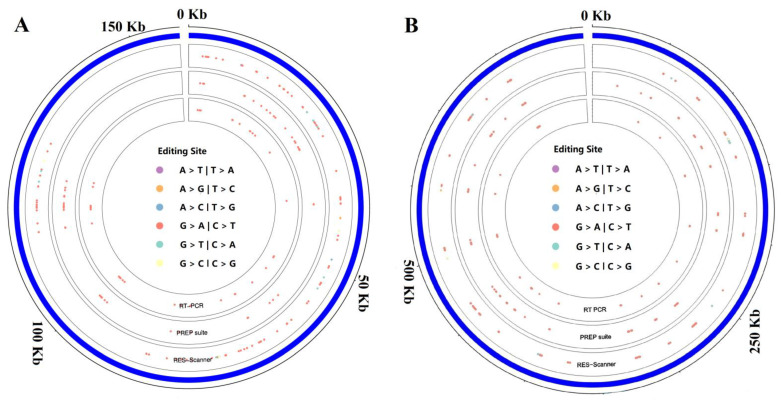
Distribution of RNA editing sites on the tea chloroplast (**A**) and mitochondria (**B**) genome. PREP suite and RES-Scanner represent the results predicted by PREP-suite and RES-Scanner, respectively. RT-PCR represents these RNA editing sites that were validated by RT-PCR.

To study RNA editing events in tea plants, potential editing sites for tea chloroplast and mitochondrial genes were predicted using PREP software (http://prep.unl.edu (accessed on 11 November 2021)) [37]. Overall, 52 and 337 RNA editing sites were predicted in the chloroplast and mitochondrial genomes (Table S2), respectively. Consistent with previous studies [11,19], these editing sites were all of the C-to-U type. In the chloroplast genome, 42 (80.77%) of the 52 sites were found at the second position of the codon, while 10 sites (19.23%) occurred at the first position (Figure 1B). Further analysis found that these chloroplast RNA editing sites resulted in 10 types of amino acid substitutions, including S > L (22), P > L (9), S > F (6), P > S (1), R > W (2), H > Y (6), L > F (1), T > I (3), A > V (1) and T > M (1) (Figure 1A). These RNA editing sites are distributed between positions 2313 and 124,204 of the chloroplast genome (Figure 2A).

For mitochondria, 65.58% (221/337) of the RNA editing sites were at the second base, while the remaining 34.42% (116/337) were at the first base (Figure 1B). Of the sites, 80 resulted in S > L transitions, followed by P > L (n = 77), S > F (n = 45), R > C (n = 29), P > S (n = 27), R > W (n = 23), H > Y (n = 20), L > F (n = 10), P > F (n = 8), T > I (n = 6), A > V (n = 5), T > M (n = 4), R > X (n = 2) and Q > X (n = 1) (Figure 1A). Surprisingly, position 718 of *atp6*, position 223 of *atp9* and position 1309 of *ccmFc* were edited to introduce a stop codon, which resulted in a premature termination of the protein translation. These RNA editing sites were mapped to positions 22,491 to 649,557 of the mitochondrial genome (Figure 2B).

To determine RNA editing events further, DNA-seq and RNA-seq data of LJ43 were applied to detect editing sites using RES-Scanner software (https://github.com/ZhangLabSZ/RES-Scanner (accessed on 1 October 2021)) [38]. A total of 125 RNA editing sites were found in the tea chloroplast genome (Table S3), 65.6% (82/125) of which were successfully annotated with BEDtools software (version 2.29.0, https://bedtools.readthedocs.io/en/latest/, accessed on 1 October 2021). Except for two editing sites in tRNA, the other 80 editing sites were annotated in protein-coding regions. A total of 35 editing sites had two types of editing, while the remaining 45 sites had only one type of editing. In mitochondria, 352 RNA editing sites were identified (Table S3), among which 227 were successfully annotated. A total of 91.2% (207/227) of the successfully annotated editing sites were in protein coding regions, and the remaining 8.8% (20/227) were in rRNA.

### 2.2. Confirmation of RNA Editing Sites in Tea Chloroplasts and Mitochondria

To confirm the RNA editing events, regions covering the editing sites were PCR-amplified using the gDNA and cDNA of LJ43 as templates. Through sequencing, 38 RNA editing sites from 22 chloroplast genes were identified and confirmed, all of which were of the C-to-U type. Among them, eight sites occurred in *ndhB*, five sites in *ndhD*, three sites in *matK*, and two sites in *atpA*, *rps2* and *rpoC2*, while the remaining 16 chloroplast genes had only one editing site (Table 1 and Figure S1). For mitochondria, 139 RNA editing sites (Table 2 and Figure S2) were detected distributing in the transcript sequences of 22 mitochondrial genes (average of 6.3 sites per gene). Among these genes, *ccmB* contained the most editing sites (n = 34), followed by *nad5* (n = 17), *cob* (n = 15), *cox2* (n = 15), *atp4* (n = 11), *atp1* (n = 6), *rpl5* (n = 6), *matR* (n = 4), *rpl10* (n = 4) and *rps12* (n = 4).

We also investigated the effects of the editing events on the codons. Of the 177 sites, 57 (51 in mitochondria and 6 in chloroplasts) located at the first position of the codon, 116 (85 in mitochondria and 31 in chloroplast) occurred at the second position and 4 (position 81 of *ndhK*, position 87 of *ccmB*, position 450 of *rpl5* and position 1722 of *matR*) occurred at the third position. Because of the above RNA editing sites, 6 and 12 types of amino acid transitions happened in chloroplasts and mitochondria, respectively (Table 1 and Table 2).

### 2.3. Sequence Features around RNA Editing Sites

**Figure 3:**
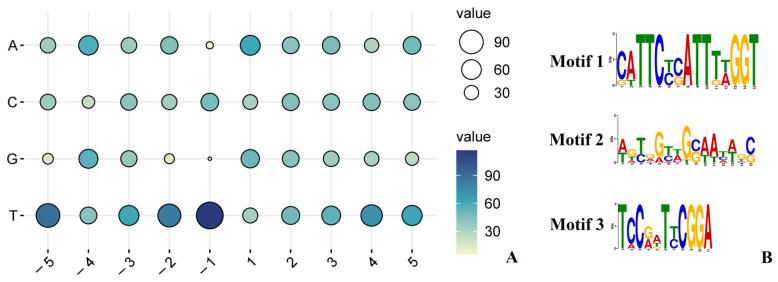
Features of sequences around RNA editing sites. (**A**) The base preference around edited C. A, C, G and T are abbreviation for nucleotide. The numbers represent the flanking base positions of the edited Cs. (**B**) Conserved motifs around RNA editing sites. The horizontal axis is the base position in the corresponding motif. The vertical axis is the fraction of bits per base.

A previous study found that the −1 position of the edited C is always T [18]. To explore the base preference around the above-described editing sites, adjacent sequences were analyzed. As shown in Figure 3A, the −5 (50.8%), −2 (48%), −1 (66.1%) and +4 (40.6%) positions upstream and downstream of the edited C frequently tended to be T. Further analysis revealed three conserved motifs in the upstream regions of 37 RNA editing sites (Figure 3B). Motifs 1 and 3 were observed upstream of six and seven RNA editing sites, respectively. The overall E-value cut-off for Motif 1 was 4.3 × 10^−4^, whereas that for Motif 3 was 1.6 × 10. Motif 2 was discovered 25 bp upstream of 23 RNA editing sites, and its overall E-value cut-off was 5.5 × 10^−4^. These three conserved motifs imply potential roles in the recognition of editing sites by RNA editing factors.

### 2.4. Impact of RNA Editing on the Subsequent Interpretation of Genetic Information

**Figure 4:**
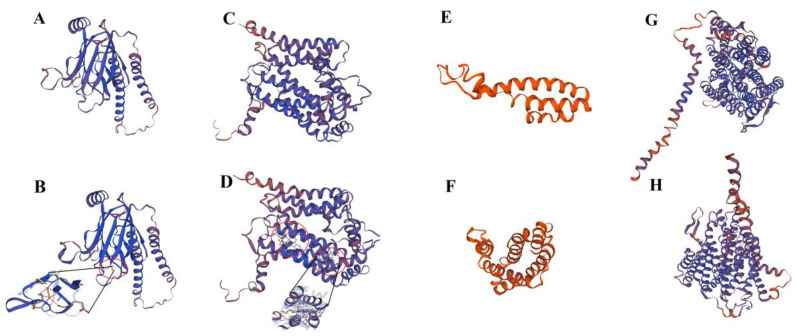
The 3-D structure of proteins were changed by RNA editing events. (**A**) Before cox2 is edited. (**B**) After cox2 is edited. Red circle indicates monomer introduced after RNA editing. (**C**) Before cob is edited. (**D**) After cob is edited. Red circles show monomers introduced after RNA editing. (**E**) Before ccmB is edited. (**F**) After ccmB is edited. (**G**) Before nad5 is edited. (**H**) After nad5 is edited.

To understand the effect of RNA editing on the target sequences, protein transmembrane domains, RNA secondary structures, protein secondary structures and partial protein 3-D structures of genes containing RNA editing sites were predicted before and after RNA editing. Although the transmembrane domains of all proteins were not altered by RNA editing (Table S4), the protein secondary structure of 16 chloroplast proteins (atpA, atpB, psbE, rpoA, psbZ, ndhF, ndhD, ndhB, ndhC, ndhH, ndhA, rps18, rps2, matK, rpoC1 and rpoC2) and 21 mitochondrial proteins except nad41 were changed, as were the RNA secondary structure of 11 chloroplasts genes (*psbE*, *rpoA*, *psbZ*, *ndhF*, *ndhD*, *ndhB*, *ndhC*, *ndhH*, *ndhA*, *rps18* and *rpoC2*) and all mitochondrial genes except *rps7*. By contrast, neither the RNA nor protein secondary structures of *psbF*, *atpF*, *rps8*, *ndhK* and *psaI* were affected by RNA editing. In addition, 3-D models of six proteins with at least eight editing sites—ndhB, nad5, atp4, cox2, cob and ccmB—were constructed using the SWISS-MODEL. Although eight RNA editing events occurred in ndhB (Figure S3A,B), the 3-D structure of its protein product was not affected. In mitochondria, the 3-D protein structure of atp4 also did not change significantly (Figure S3C,D). However, RNA editing of cox2 introduced a DINUCLEAR COPPER ION monomer (Figure 4A,B). In cob, two PROTOPORPHYRIN IX CONTAINING FE monomers were generated by RNA editing (Figure 4C,D), and there were only five α-helices before ccmB editing, compared to nine α-helices and two β-sheets thereafter (Figure 4E,F). nad5 gained one α-helix but lost two β-sheets after RNA editing (Figure 4G,H). These results imply that the function of organelle proteins might be affected by RNA editing sites.

### 2.5. Relationship between RNA Editing and Etiolation and Albino Tea Plants

**Figure 5:**
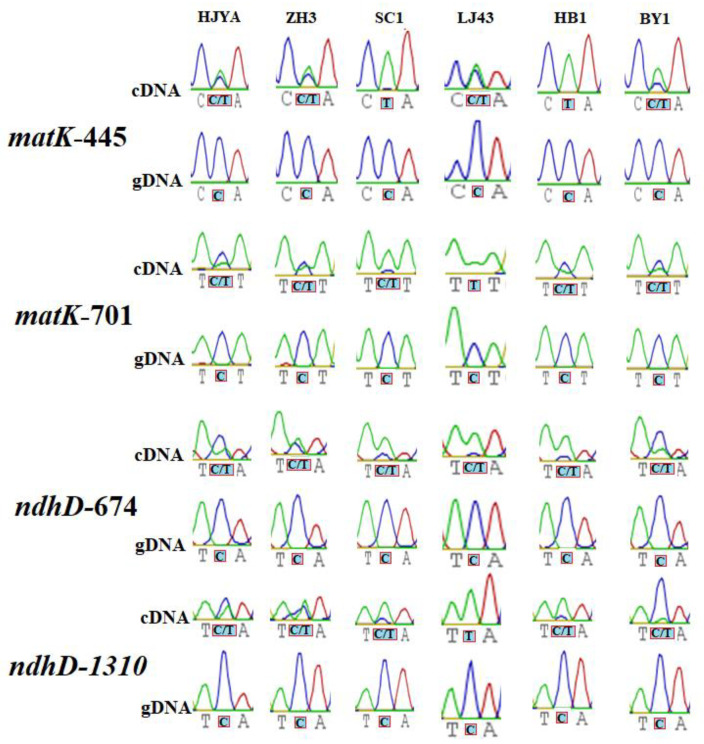
Editing levels of 4 RNA editing events in 6 varieties (LJ43 and SC1 with normal green leaves, HJYA and ZH3 with etiolation leaves and HB1 and BY1 with albino leaves) of tea plants. HJYA represents *C. sinensis cv. ‘Huangjinya’*; ZH3 represents *C. sinensis cv. ‘Zhonghuang 3’*; SC1 represents *C. sinensis cv. ‘Shaancha 1’*; LJ43 represents *C. sinensis cv. ‘Longjing 43’*; HB1 represents *C. sinensis cv. ‘Huabai 1’*; BY1 represents *C. sinensis cv. ‘Baiye 1’*. The *matk-445* represents 445 position of *matK*. The *matk-701* represents 701 position of *matK*. The *ndhD-674* represents 674 position of *ndhD*. The *ndhD-1310* represents 1310 position of *ndhD*. Red boxes indicate the RNA editing sites in the gDNA and cDNA.

Previous reports found that RNA editing events were associated with leaf color changes in plants [12,30]. To explore the potential relationship between RNA editing and tea leaf color changes, editing levels of *matK-445* (position 445 of *matK*), *matK-701*, *ndhD-674* and *ndhD-1310* sites in different cultivars (including LJ43 and SC1 with normal green leaves, HJYA and ZH3 with etiolation leaves and HB1 and BY1 with albino leaves) were analyzed. As shown in Figure 5, the ratio of the T peak to the sum of the C and T peak heights in the sequencing chromatogram represents the level of editing in the individual transcripts. The editing level of *matK-445* was over 90% in SC1 and HB1, around 65% in BY1 and slightly more than 50% in LJ43, HJYA and ZH3. The editing extent of *matK-701* was approximately 30% in the albino cultivars BY1 and HB1, and about 30% and 45% in etiolation cultivars HJYA and ZH3, respectively, whereas it exceeded 95% in green varieties LJ43 and SC1. In LJ43, SC1 and HB1, more than 80% of *ndhD-674* was edited, whereas about half C was edited in ZH3 and around 30% in BY1 and HJYA. The extent of editing of *ndhD-1310* was completely edited in LJ43, ~80% in SC1 and HB1, ~50% in ZH3, ~40% in HJYA and <10% in BY1. These results indicated that the levels of RNA editing might have some physiological connection with the color change of tea leaves.

### 2.6. Interaction Prediction of PLS-CsPPR and the Target Sequences of RNA Editing Sites

Numerous studies indicate that PLS-PPR proteins are important RNA editing factors [2,3], and 295 PLS-CsPPR proteins were found in tea plants [36]. Therefore, we used the method of Kobayashi et al. (see material and methods) to analyze the possible interactions between the PLS-CsPPR proteins and target sequences containing validated RNA editing sites. As shown in Table S6, 159 of the 177 target sequences were predicted to interact with 218 PLS-CsPPR proteins. Among them, 36.7% of PLS-CsPPR proteins had only one target sequence; 25.2% had two target sequences; 19.7% had three target sequences; 8.3% had four target sequences; and 10% had ≥5 target sequences. The potential interactions between PLS-CsPPR proteins and target sequences implied that the PLS-CsPPR proteins might be involved in the recognition of these RNA editing sites.

## 3. Discussion

RNA editing seemed to be a challenge to the central dogma of molecular biology at the transcriptional level, and has received increasing attention [2,3]. In recent years, a large number of RNA editing factors were reported to be involved in the C-to-U deamination reaction [39,40,41], and many studies attempted to use expressed sequence tags (EST) sequences [42] or high-throughput sequencing technology to identify additional RNA editing sites in plants [43,44]. Moreover, in order to distinguish the changed sites due to heteroplasmy or the RNA editing events, total RNA-seq as well as DNA-seq was used for analysis [45], but the heteroplasmy could not be excluded in this work.

In this study, we used two software packages to investigate RNA editing sites. PREP predicted 52 and 337 RNA editing sites in the tea plant chloroplast and mitochondrial genomes, respectively (Figure 2), while RES-Scanner software (https://github.com/ZhangLabSZ/RES-Scanner (accessed on 1 October 2021)) identified 125 and 300 sites, respectively. One reason for the different numbers of editing sites was that PREP could only predict RNA editing sites in the protein-coding regions of 35 chloroplast genes and 43 mitochondrial genes; another was that RES-Scanner could cause read mismatches, resulting in false-positive editing sites. Therefore, PCR amplification with gDNA and cDNA templates was performed to verify the combined predicted results. In total, 38 and 139 C-to-U RNA editing events were found in the protein-coding regions of chloroplast and mitochondrial genes, respectively. Similar to previous studies [2,3], the C-to-U type is the main form of RNA editing in tea plants. Although this method is widely used to identify RNA editing sites, we cannot exclude that these editing sites are caused by heterogeneity [45].

Previous studies suggested that RNA editing events mostly occurred at the first or second position of a codon [2,11]. However, we found four RNA editing sites located at the third position of the codon, similar to *Sweet Sorghum* [46]. A study in Arabidopsis noted a higher editing level of base C when the adjacent −1 position was T, whereas a lower editing level was seen when the −1 position was G [18]. In the −1 position of the validated RNA editing sites in this study, T was more frequent (Figure 3A), similar to *A. thaliana*. Generally, 20–25 nucleotides upstream of the RNA editing site are involved in the binding of editing factors [47]. Consistent with previous results for *S. miltiorrhiza* [21], analysis with MEME software (http://meme-suite.org/tools/meme (accessed on 19 March 2022)) found three conserved motifs (with lengths of 15, 15 and 11 nucleotides) in tea plants (Figure 3B). These motifs might be involved in the recognition of editing sites by RNA editing factors.

RNA editing changes the nucleotide at the corresponding position [2,11] and alters the transmembrane domain and number of alpha helices. However, we found no changes in the protein transmembrane domain before and after RNA editing in tea plants, possibly because RNA editing events rarely or never occurred in the transmembrane region. Similar to previous findings [18], our prediction data showed that the RNA secondary structure of 32 genes and protein secondary structures of 37 genes were affected by RNA editing. In addition, 3-D structure analysis showed that the 3-D structures of five mitochondrial proteins (nad5, atp4, cox2, cob and ccmB) were altered after RNA editing. Abnormal editing of mitochondrial genes can lead to a pale green leaf phenotype [46]. Therefore, RNA editing seems to be tightly associated with the color change of plant leaves.

To date, a total of five types of RNA editing factors were discovered, among which the PLS-PPR protein is considered to be the most important and has been extensively studied [2,3]. The second, fifth, and last positions of the PPR motif play an important role in the recognition of RNA bases by the PPR motif [23,48] and can help the PPR motif recognize RNA bases [49,50]. However, a recent study noted that using the second, fifth and last amino acids of the PPR motif to predict downstream target sequences containing RNA editing sites would obtain more accurate results [51]. An absence of RNA editing can cause albino leaves and etiolation in some Arabidopsis and rice mutants, such as *atclb19*, *osppr6*, *osdua1* and *atgun1* [12,13,30,52], and many albino and etiolation varieties of tea plants have been bred [32], such as Baiye 1, Huabai 1, Huangjinya, etc. However, whether the extent of RNA editing is affected by the color change of tea leaves remains unclear. We investigated the extent of editing of four RNA editing sites in six varieties. Most notably, *matK-701* was edited more than 90% in green varieties (SC1 and LJ43), whereas it was about 30% in albino varieties (BY1 and HB1) and no more than 50% in etiolation varieties (HJYA and ZH3). A previous study found that OTP81 (QED1) plays a very important role in the editing of *matK-706* [53], and it was found to be associated with the color change of plant leaves [52,54]. Perhaps a similar gene exists in the tea plants; it is our next research focus.

## 4. Materials and Methods

### 4.1. Plant Materials and Growth Conditions

All tea tree cuttings (*C. sinensis cv. ‘Longjing 43’*, LJ43; *C. sinensis cv. ‘Shaancha 1’*, SC1; *C. sinensis cv. ‘Huangjinya’*, HJYA; *C. sinensis cv. ‘Zhonghuang 3’*, ZH3; *C. sinensis cv. ‘Huabai 1’*, HB1; *C. sinensis cv. ‘Baiye 1’*, BY1) used in this study were planted in the greenhouse of Northwest A&F University (Yangling, China) under natural light, 20 °C to 25 °C ambient temperature and 60% ± 10% relative humidity. The normal green leaves of LJ43 and SC1, etiolation leaves of HJYA and ZH3, albino leaves of HB1 and BY1 were collected on 5 July 2021, respectively. The fresh leaf samples were immediately snap-frozen in liquid nitrogen and stored in a −80 °C freezer for DNA and RNA extraction.

### 4.2. Data Collection

The chloroplast genome and General Feature Format (GFF) of LJ43 were downloaded from the Tea Plant Genome Database (TPGD, http://eplant.njau.edu.cn/tea/index.html (accessed on 1 October 2021)). The annotation information for the chloroplast genome of *C. sinensis* was downloaded from The Rocap Lab (https://rocaplab.ocean.washington.edu/tools/cpbase/ (accessed on 1 October 2021)) and then integrated with the GFF file to generate a new complete chloroplast genome annotation file of LJ43. The mitochondria genome and GFF of *C. sinensis var Assamica* were downloaded from TPGD (http://eplant.njau.edu.cn/tea/index.html (accessed on 1 October 2021)). The DNA-seq (SRR12333861, SRR1227251 and SRR12350542) and RNA-seq (ERR4369160, ERR4369193, ERR4369194, ERR4369195, ERR4369196, ERR4369197, ERR4369198, ERR4369199, ERR4369200, ERR4369205, ERR4369206 and ERR4369207) data of LJ43 [55] were downloaded from the National Center for Biotechnology Information Sequence Read Archive (NCBI-SRA, https://www.ncbi.nlm.nih.gov/ (accessed on 15 October 2021)) and European Bioinformatics Institute (https://www.ebi.ac.uk/ena/browser/home (accessed on 15 October 2021)), respectively.

### 4.3. Identification of RNA Editing Sites in Tea Chloroplasts and Mitochondria

The RNA editing sites were identified using the Predictive RNA Editor for Plants suite (PREP-suite, http://prep.unl.edu (accessed on 11 November 2021)) [37]. DNA-seq and RNA-seq of LJ43 were used to determine RNA editing sites further. Firstly, the trimmomatic (Version 0.39) [56] was used to filter low-quality reads in DNA-seq and RNA-seq with the following parameters: LEADING and TRAILING: 3, MINLEN: 51. Then, the FastQC (Version 0.11.9, https://www.bioinformatics.babraham.ac.uk/projects/fastqc/ (accessed on 1 October 2021)) was used to check the quality of the reads. Finally, the RES-Scanner [38] was used to identify RNA editing sites using the chloroplast genome of LJ43 and the mitochondrial genome of *C. sinensis var. Assamica* as reference sequences. After that, RNA editing sites were annotated using bedtools [57] according to gene features.

### 4.4. Validation of Predicted RNA Editing Sites

The tea leaves were divided into two parts along the main vein of the leaves. Half of them were used to extract gDNA (RNA free) using a Plant Genomic DNA Isolation Kit (Tsingke, Beijing, China). The total RNA was extracted from the other half of the samples using a Plant Total RNA Isolation Kit (Vazyme, Nanjing, China). A total of 1% agarose gel electrophoresis was used to check the integrity of total RNA. The concentration of total RNA was measured with a NanoDrop ND 1000 spectrophotometer (Thermo Fisher Scientific, Waltham, MA, USA). A total of 1–2 μg of the total RNA was used to synthesize the first-strand complementary DNA (cDNA) with HiScript Q RT SuperMix for qPCR (+gDNA wiper) (Vazyme, Nanjing, China). Specific primers were used to amplify genes containing RNA editing sites using the gDNA and cDNA as templates, respectively (Table S1).

### 4.5. Contextual and Potential Motifs Analysis around RNA Editing Sites

To study the upstream and downstream bases’ preference of RNA editing sites, 10 bases (except position 0) from the position −5 to +5 of the editing site were extracted. In addition, the upstream 25 bases of the RNA editing site were used to discover potential binding motifs for RNA editing factors using Multiple Em for Motif Elicitation [58] (MEME, http://meme-suite.org/tools/meme (accessed on 19 March 2022)).

### 4.6. Structural Analysis of Proteins and RNAs

The RNAfold [59] (http://rna.tbi.univie.ac.at/cgi-bin/RNAWebSuite/RNAfold.cgi (accessed on 19 February 2022)) web server was employed to predict changes in the RNA secondary structure with or without RNA editing using default parameters. Effects of RNA editing events on the transmembrane domain and the secondary structure of the protein were investigated using the TMHMM tool [60] (https://services.healthtech.dtu.dk/service.php?TMHMM-2.0 (accessed on 19 February 2022)) and PSIPRED [61] (http://bioinf.cs.ucl.ac.uk/psipred/ (accessed on 21 February 2022)), respectively. The three-dimensional structural model of the protein was constructed using the SWISS-MODEL [62] (https://swissmodel.expasy.org/interactive (accessed on 21 February 2022)).

### 4.7. Interaction Analysis between PLS-CsPPR Proteins and Target Sequence Containing RNA Editing Sites

Interaction analysis between the PLS-CsPPR protein and their target sequence was performed according to the method of Kobayashi et al. [49] with minor modifications. In brief, the PPR motifs of PLS-CsPPR were obtained from our previous study [36]; the second, fifth and last amino acid residues of these PPR motifs were extracted to form 3-, 2- and 1-letter codes (Table S5). The PPR code dataset of Kobayashi et al. [49] was used to compose the base preference matrix of PLS-CsPPR proteins. The 51 bases surrounding the RNA editing site (position −25 to position +25) were used as target sequences. The PPR matrices and target sequences as queries to the Find Individual Motif Occurrences [63] (FIMO, https://meme-suite.org/meme/tools/fimo (accessed on 13 April 2022)) program in the MEME suite software (https://meme-suite.org/meme (accessed on 13 April 2022)) in the MEME suite.

## 5. Conclusions

RNA editing is an important post-transcriptional process, which alters the nucleotide sequences of RNA, such that the genetic information and phenotype of plants are changed. PPR proteins widely exist in plants and are dominantly localized in plastids and mitochondria, which play essential roles in organellar RNA metabolism. This study systematically analyzed RNA editing events in tea chloroplast and mitochondria genomes; 38 and 139 RNA editing events were validated by PCR and sequence analysis. Analysis of the base preference around the RNA editing sites showed that in the −1 position, C to T were more frequent occurrences. Structural analyses indicated that RNA the secondary structure of 32 genes, protein secondary structure of 37 genes and the 3-D structure of 5 proteins were altered due to RNA editing. The editing level analysis of 4 RNA editing sites (*matK-445*, *matK-701*, *ndhD-674* and *ndhD-1310*) in 6 cultivars (2 green varieties, 2 albino varieties and 2 etiolation varieties) indicated that *matK-701* might be involved in the color change of tea leaves. Furthermore, the interaction between 159 target sequences and 218 PLS-PPR proteins were predicted through PPR-RNA code. These data provide new insights into of RNA editing phenomenon of the tea plant, which will facilitate further study of albino and etiolation in tea leaves.

## Figures and Tables

**Table 1:** RNA editing sites in tea chloroplast.

Gene	Genome Position	Edited Nucleotide	Amino Acid Changes	Editing Type	Position in Codon
*matK*	2313	C1234	H412→Y412	C→T	1
*matK*	2846	C701	S234→F234	C→T	2
*matK*	3102	C445	H149→Y149	C→T	1
*atpA*	11,605	C914	S305→L305	C→T	2
*atpA*	11,728	C791	P264→L264	C→T	2
*atpF*	13,757	C92	P31→H31	C→T	2
*rps2*	16,983	C248	S83→L83	C→T	2
*rps2*	17,097	C134	T45→I45	C→T	2
*rpoC2*	17,886	C3728	S1243→L1243	C→T	2
*rpoC2*	18,765	C2849	S950→F950	C→T	2
*rpoC1*	24,531	C62	S21→L21	C→T	2
*psbZ*	37,856	C50	S17→L17	C→T	2
*ndhK*	52,675	C81	F27→F27	C→T	3
*ndhC*	52,958	C40	L14→L14	C→T	1
*atpB*	56,330	C23	S8→F8	C→T	2
*psaI*	61,448	C80	S27→F27	C→T	2
*psbF*	66,719	C77	S26→F26	C→T	2
*psbE*	66,843	C214	P72→S72	C→T	1
*rps18*	70,747	C221	S74→L74	C→T	2
*petB*	78,764	C611	P204→L204	C→T	2
*rpoA*	81,234	C200	S67→F67	C→T	2
*rps8*	82,841	C182	P61→L61	C→T	2
*ndhB*	97,156	C1481	P494→L494	C→T	2
*ndhB*	97,807	C830	S277→L277	C→T	2
*ndhB*	98,570	C746	S249→F249	C→T	2
*ndhB*	98,579	C737	P246→L246	C→T	2
*ndhB*	98,705	C611	S204→L204	C→T	2
*ndhB*	98,729	C586	H196→Y196	C→T	1
*ndhB*	98,848	C467	P156→L156	C→T	2
*ndhB*	99,167	C149	S50→L50	C→T	2
*ndhF*	114,736	C290	S97→L97	C→T	2
*ndhD*	118,369	C1310	S437→L437	C→T	2
*ndhD*	118,792	C887	P296→L296	C→T	2
*ndhD*	118,801	C878	S293→L293	C→T	2
*ndhD*	119,005	C674	S225→L225	C→T	2
*ndhD*	119,296	C383	S128→L128	C→T	2
*ndhA*	124,204	C341	S114→L114	C→T	2
*ndhH*	125,201	C505	H169→Y169	C→T	1

**Table 2:** RNA editing site in tea mitochondria.

Gene	Genome Position	Edited Nucleotide	Amino Acid Changes	Editing Type	Position in Codon
*atp9*	54,812	C20	S7→L7	C→T	2
*atp9*	54,874	C82	L28→F28	C→T	1
*atp9*	54,884	C92	S31→L31	C→T	2
*nad6*	85,722	C446	S152→F152	C→T	2
*cox2*	138,085	C742	R248→W248	C→T	1
*cox2*	138,129	C698	T233→M233	C→T	2
*cox2*	138,195	C632	S211→L211	C→T	2
*cox2*	138,246	C581	S194→L194	C→T	2
*cox2*	138,270	C557	P186→L186	C→T	2
*cox2*	138,283	C544	P182→S182	C→T	1
*cox2*	138,351	C476	S159→L159	C→T	2
*cox2*	138,366	C461	P154→L154	C→T	2
*cox2*	138,384	C443	T148→M148	C→T	2
*cox2*	138,448	C379	R127→W127	C→T	1
*cox2*	138,549	C278	P93→L93	C→T	2
*cox2*	138,574	C253	R85→W85	C→T	1
*cox2*	138,664	C163	R55→W55	C→T	1
*cox2*	138,666	C161	S54→L54	C→T	2
*cox2*	138,756	C71	S24→F24	C→T	2
*rps12*	196,313	C284	S95→F95	C→T	2
*rps12*	196,401	C196	H66→Y66	C→T	1
*rps12*	196,497	C100	R34→C34	C→T	1
*rps12*	196,526	C71	S24→L24	C→T	2
*nad3*	196,959	C43	P15→S15	C→T	1
*rps1*	239,984	C107	S36→F36	C→T	2
*rps1*	240,035	C56	P19→L19	C→T	2
*matR*	272,897	C1775	P592→L592	C→T	2
*matR*	272,950	C1722	Y574→Y574	C→T	3
*matR*	272,984	C1688	P563→L563	C→T	2
*matR*	273,005	C1667	S556→F556	C→T	2
*ccmFn*	297,223	C1423	R475→W475	C→T	1
*ccmFn*	297,286	C1486	L496→F496	C→T	1
*ccmFn*	297,355	C1555	P519→S519	C→T	1
*atp4*	323,099	C416	T139→I139	C→T	2
*atp4*	323,109	C406	P136→S136	C→T	1
*atp4*	323,120	C395	S132→L132	C→T	2
*atp4*	323,264	C251	P84→L84	C→T	2
*atp4*	323,267	C248	P83→L83	C→T	2
*atp4*	323,288	C227	P76→L76	C→T	2
*atp4*	323,300	C215	S72→L72	C→T	2
*atp4*	323,397	C118	R40→C40	C→T	1
*atp4*	323,426	C89	S30→L30	C→T	2
*atp4*	323,444	C71	S24→L24	C→T	2
*atp4*	323,456	C59	S20→F20	C→T	2
*nad41*	323,824	C149	S50→L50	C→T	2
*nad41*	323,872	C101	S34→L34	C→T	2
*atp1*	396,108	C1039	P347→S347	C→T	1
*atp1*	396,133	C1064	S355→L355	C→T	2
*atp1*	396,247	C1178	S393→L393	C→T	2
*atp1*	396,285	C1216	L406→F406	C→T	1
*atp1*	396,361	C1292	P431→L431	C→T	2
*atp1*	396,484	C1415	P472→L472	C→T	2
*rps7*	421,118	C332	S111→L111	C→T	2
*rpl10*	431,602	C83	S28→L28	C→T	2
*rpl10*	431,620	C101	S34→L34	C→T	2
*rpl10*	431,758	C239	S80→L80	C→T	2
*rpl10*	431,833	C314	S105→L105	C→T	2
*ccmB*	432,360	C554	S185→L185	C→T	2
*ccmB*	432,363	C551	S184→L184	C→T	2
*ccmB*	432,400	C514	R172→C172	C→T	1
*ccmB*	432,402	C512	S171→F171	C→T	2
*ccmB*	432,411	C503	P168→L168	C→T	2
*ccmB*	432,420	C494	S165→L165	C→T	2
*ccmB*	432,429	C485	S162→L162	C→T	2
*ccmB*	432,438	C476	P159→L159	C→T	2
*ccmB*	432,439	C475	P159→L159	C→T	1
*ccmB*	432,477	C467	S156→L156	C→T	2
*ccmB*	432,486	C428	S143→L143	C→T	2
*ccmB*	432,490	C424	R142→C142	C→T	1
*ccmB*	432,535	C379	L127→L127	C→T	1
*ccmB*	432,547	C367	R123→W123	C→T	1
*ccmB*	432,576	C338	P113→L113	C→T	2
*ccmB*	432,601	C313	R105→W105	C→T	1
*ccmB*	432,610	C304	R102→C102	C→T	1
*ccmB*	432,628	C286	R96→W96	C→T	1
*ccmB*	432,720	C194	P65→F65	C→T	2
*ccmB*	432,721	C193	P65→F65	C→T	1
*ccmB*	432,735	C179	P60→L60	C→T	2
*ccmB*	432,742	C172	P58→S58	C→T	1
*ccmB*	432,750	C164	P55→L55	C→T	2
*ccmB*	432,754	C160	P54→S54	C→T	1
*ccmB*	432,760	C154	R52→W52	C→T	1
*ccmB*	432,765	C149	P50→L50	C→T	2
*ccmB*	432,766	C148	P50→L50	C→T	1
*ccmB*	432,777	C137	S46→F46	C→T	2
*ccmB*	432,786	C128	S43→L43	C→T	2
*ccmB*	432,827	C87	I29→I29	C→T	3
*ccmB*	432,834	C80	S27→L27	C→T	2
*ccmB*	432,843	C71	P24→L24	C→T	2
*ccmB*	432,871	C43	P15→S15	C→T	1
*ccmB*	432,886	C28	S10→L10	C→T	1
*atp6*	450,313	C548	S183→F183	C→T	2
*atp6*	450,607	C254	S85→L85	C→T	2
*atp6*	450,824	C37	P12→S12	C→T	1
*nad5*	504,064	C155	P52→L52	C→T	2
*nad5*	505,005	C245	P82→L82	C→T	2
*nad5*	505,035	C275	S92→F92	C→T	2
*nad5*	505,121	C361	P121→F121	C→T	1
*nad5*	505,122	C362	P121→F121	C→T	2
*nad5*	505,137	C377	P126→L126	C→T	2
*nad5*	505,161	C401	S134→F134	C→T	2
*nad5*	505,269	C509	P170→L170	C→T	2
*nad5*	505,302	C542	P181→L181	C→T	2
*nad5*	505,311	C551	S184→L184	C→T	2
*nad5*	505,371	C611	A204→V204	C→T	2
*nad5*	505,392	C632	S211→F211	C→T	2
*nad5*	505,394	C634	R212→C212	C→T	1
*nad5*	505,439	C679	L227→F227	C→T	1
*nad5*	505,476	C716	S239→L239	C→T	2
*nad5*	505,488	C728	S243→L243	C→T	2
*nad5*	505,598	C838	P280→S280	C→T	1
*orf115b*	506,667	C77	S50→L50	C→T	2
*ccmFc*	553,661	C1133	P378→L378	C→T	2
*ccmFc*	553,682	C1154	S385→L385	C→T	2
*ccmFc*	553,790	C1262	S421→L421	C→T	2
*sdh3*	576,179	C67	P23→S23	C→T	1
*sdh3*	576,186	C74	S25→F25	C→T	2
*rpl5*	646,335	C92	S31→L31	C→T	2
*rpl5*	646,412	C169	P57→S57	C→T	1
*rpl5*	646,415	C172	R58→C58	C→T	1
*rpl5*	646,693	C450	I150→I150	C→T	3
*rpl5*	646,761	C518	P173→L173	C→T	2
*rpl5*	646,764	C521	P174→L174	C→T	2
*rps14*	647,079	C271	P91→S91	C→T	1
*cob*	648,551	C118	P40→S40	C→T	1
*cob*	648,611	C178	H60→Y60	C→T	1
*cob*	648,719	C286	L96→F96	C→T	1
*cob*	648,731	C298	H100→Y100	C→T	1
*cob*	648,758	C325	H109→Y109	C→T	1
*cob*	648,791	C358	R120→W120	C→T	1
*cob*	648,852	C419	P140→L140	C→T	2
*cob*	649,001	C568	H150→Y150	C→T	1
*cob*	649,113	C680	S227→F227	C→T	2
*cob*	649,241	C808	P270→S270	C→T	1
*cob*	649,286	C853	H285→Y285	C→T	1
*cob*	649,341	C908	P303→L303	C→T	2
*cob*	649,347	C914	S305→F305	C→T	2
*cob*	649,415	C982	H327→Y327	C→T	1
*cob*	649,448	C1015	R339→C339	C→T	1

## Data Availability

The data supporting the findings of this study are available in the article and its supplementary materials. The publicly data, DNA-seq (SRR12333861, SRR1227251 and SRR12350542) and RNA-seq (ERR4369160, ERR4369193, ERR4369194, ERR4369195, ERR4369196, ERR4369197, ERR4369198, ERR4369199, ERR4369200, ERR4369205, ERR4369206 and ERR4369207) data of LJ43 [55], were downloaded from the National Center for Biotechnology Information Sequence Read Archive (NCBI-SRA, https://www.ncbi.nlm.nih.gov/ (accessed on 15 October 2021)) and European Bioinformatics Institute (https://www.ebi.ac.uk/ena/browser/home (accessed on 15 October 2021)), respectively.

## Supplementary Materials

The following supporting information can be downloaded at: https://www.mdpi.com/article/10.3390/ijms232113640/s1.
