# Supplementary material for: Exploring the RNA Editing Events and Their Potential Regulatory Roles in Tea Plant (Camellia sinensis L.)
Source: Int J Mol Sci. 2022 Nov 7;23(21):13640. doi: 10.3390/ijms232113640 (PMC9654872; doi:10.3390/ijms232113640)
Supplement: Supplementary file 1 [file ijms-23-13640-s001.zip › Figures S.pdf]

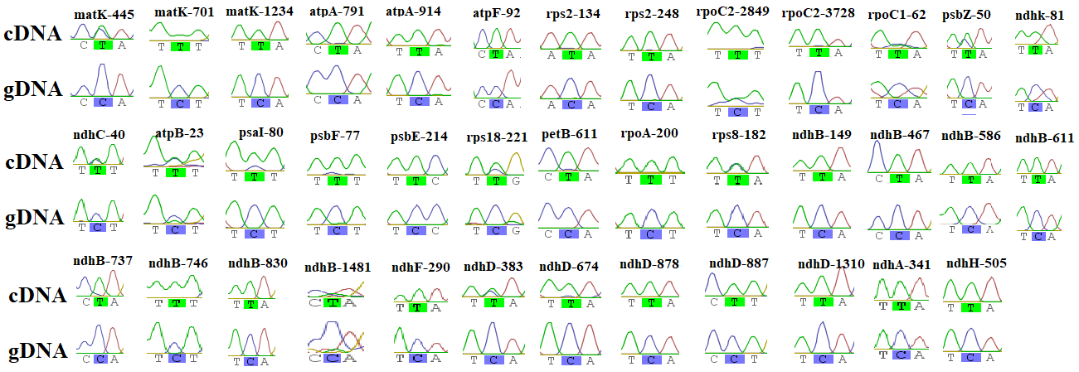

Figure S1. Sequencing chromatograms show RNA-editing sites in tea chloroplast.

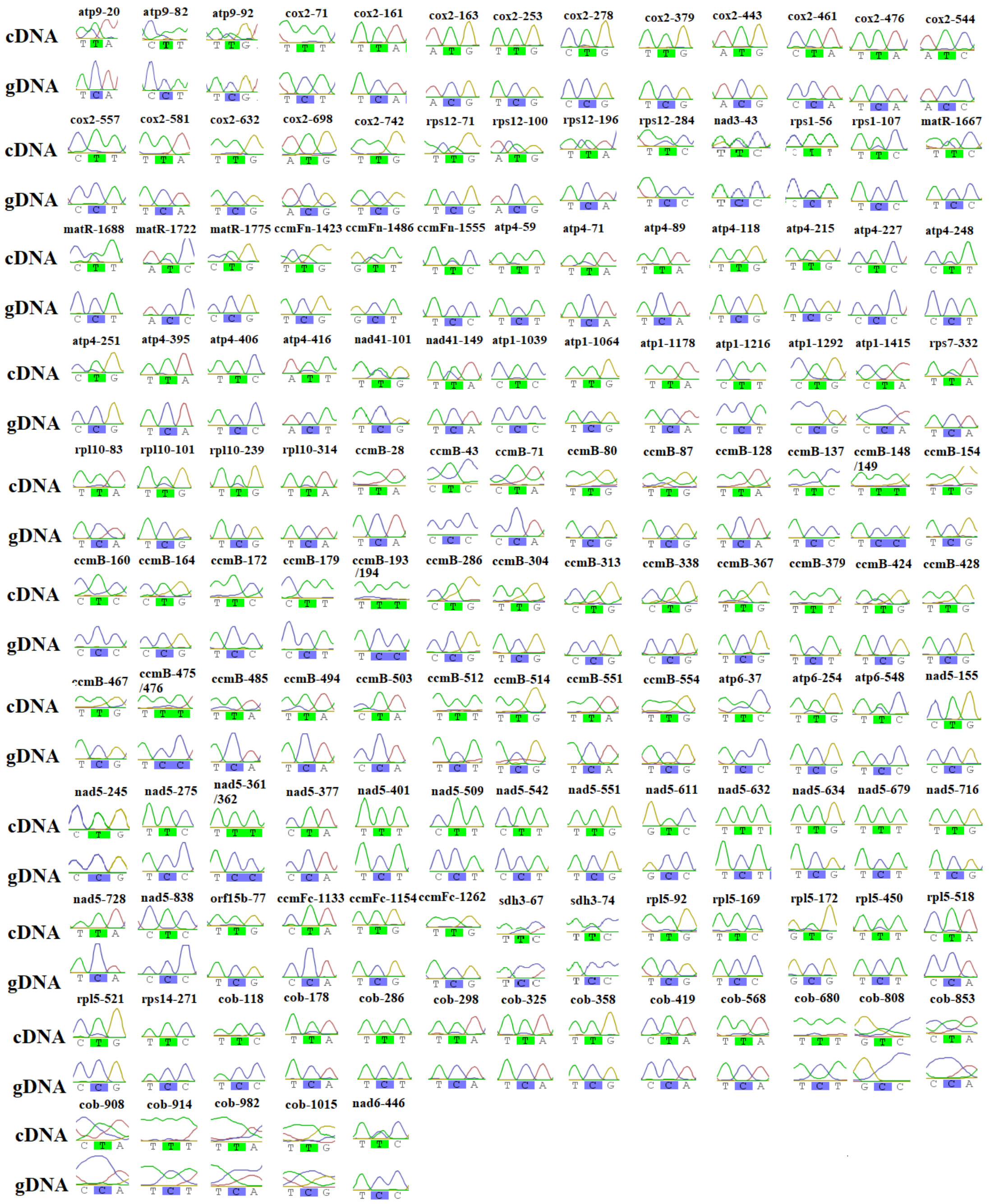

Figure S2. Sequencing chromatograms show RNA-editing sites in tea mitochondria.

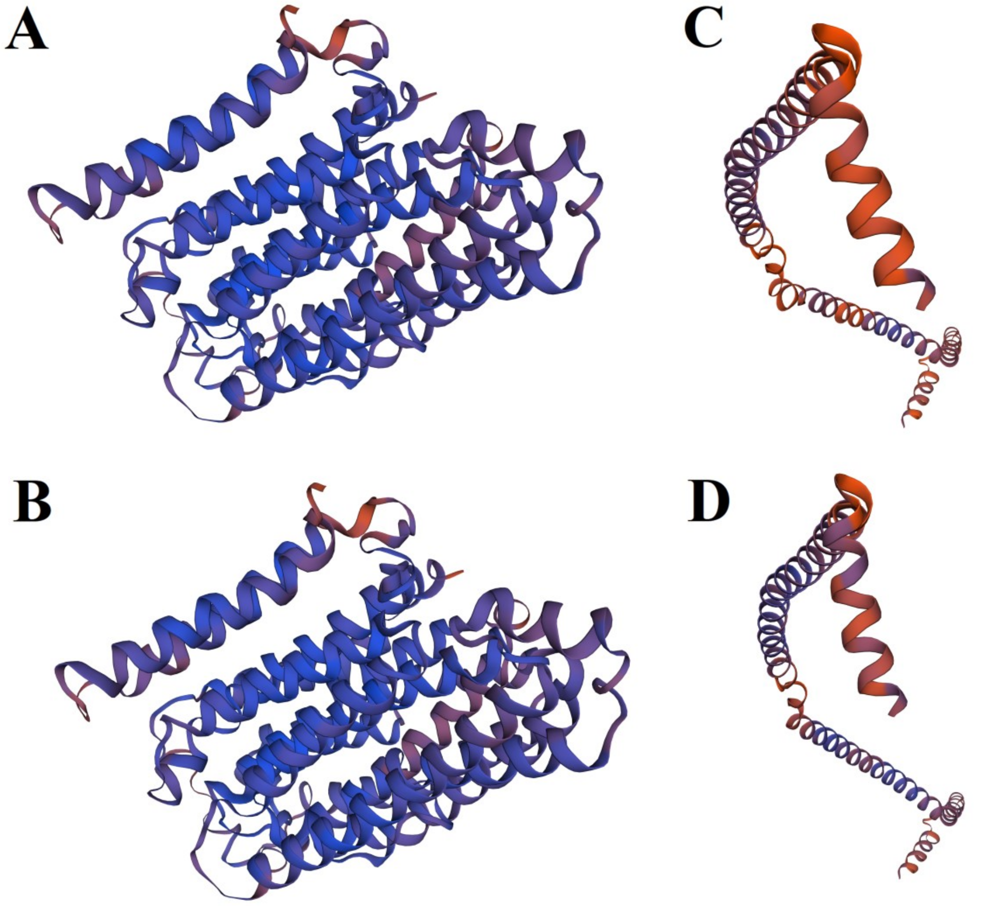

Figure S3. 3-D structures of ndhB and atp4. (A) Before ndhB is edited. (B) After ndhB is edited. (C) Before atp4 is edited. (D) After atp4 is edited.
